# Distinct and Overlapping Effector Functions of Expanded Human CD4^+^, CD8α^+^ and CD4^-^CD8α^-^ Invariant Natural Killer T Cells

**DOI:** 10.1371/journal.pone.0028648

**Published:** 2011-12-12

**Authors:** Vincent O'Reilly, Shijuan G. Zeng, Gabriel Bricard, Ann Atzberger, Andrew E. Hogan, John Jackson, Conleth Feighery, Steven A. Porcelli, Derek G. Doherty

**Affiliations:** 1 Department of Immunology and Institute of Molecular Medicine, Trinity College Dublin, St. James's Hospital, Dublin, Ireland; 2 Department of Microbiology and Immunology, Albert Einstein College of Medicine, Bronx, New York, United States of America; 3 Obesity Immunology Group, Education and Research Centre, St. Vincent's University Hospital and University College Dublin, Dublin, Ireland; Karolinska Institutet, Sweden

## Abstract

CD1d-restricted invariant natural killer T (iNKT) cells have diverse immune stimulatory/regulatory activities through their ability to release cytokines and to kill or transactivate other cells. Activation of iNKT cells can protect against multiple diseases in mice but clinical trials in humans have had limited impact. Clinical studies to date have targeted polyclonal mixtures of iNKT cells and we proposed that their subset compositions will influence therapeutic outcomes. We sorted and expanded iNKT cells from healthy donors and compared the phenotypes, cytotoxic activities and cytokine profiles of the CD4^+^, CD8α^+^ and CD4^−^CD8α^−^ double-negative (DN) subsets. CD4^+^ iNKT cells expanded more readily than CD8α^+^ and DN iNKT cells upon mitogen stimulation. CD8α^+^ and DN iNKT cells most frequently expressed CD56, CD161 and NKG2D and most potently killed CD1d^+^ cell lines and primary leukemia cells. All iNKT subsets released Th1 (IFN-γ and TNF-α) and Th2 (IL-4, IL-5 and IL-13) cytokines. Relative amounts followed a CD8α>DN>CD4 pattern for Th1 and CD4>DN>CD8α for Th2. All iNKT subsets could simultaneously produce IFN-γ and IL-4, but single-positivity for IFN-γ or IL-4 was strikingly rare in CD4^+^ and CD8α^+^ fractions, respectively. Only CD4^+^ iNKT cells produced IL-9 and IL-10; DN cells released IL-17; and none produced IL-22. All iNKT subsets upregulated CD40L upon glycolipid stimulation and induced IL-10 and IL-12 secretion by dendritic cells. Thus, subset composition of iNKT cells is a major determinant of function. Use of enriched CD8α^+^, DN or CD4^+^ iNKT cells may optimally harness the immunoregulatory properties of iNKT cells for treatment of disease.

## Introduction

Invariant natural killer T (iNKT) cells are cytotoxic T lymphocytes that express NK cell markers and a T cell receptor (TCR) composed of an invariant α-chain (Vα24Jα18 in humans and Vα14Jα18 in mice) paired with one of a limited number of β-chains. iNKT cells recognize glycolipid antigens presented by the major histocompatibility complex class I-like molecule CD1d [1], [2]. They can recognize a number of self and bacterial glycolipids [3], [4] but the most potent activator of iNKT cells known to date is the marine sponge-derived glycolipid α-galactosylceramide (α-GalCer) [5]. Upon activation with α-GalCer, iNKT cells can kill a wide range of tumor cell lines [6], [7] and secrete a diverse range of growth factors and cytokines that activate and polarize adaptive immune responses [1], [2], [8]–[12]. Activated iNKT cells can also interact directly with other cells of the immune system and can induce the maturation of dendritic cells (DC) into antigen-presenting cells (APC) [13]–[15] and of B cells into antibody-secreting plasma cells [16], [17]. Therapeutic activation of iNKT cells in murine models can prevent tumor growth, ameliorate autoimmune disease and protect against microbial infection [6], [18]–[20]. Numerical and functional iNKT cell deficiencies have been reported in a number of human diseases [21]–[25], but clinical trials that have targeted iNKT cells in humans have to date been somewhat disappointing [26]–[30].

A reason for the low efficacy of iNKT cells in human immunotherapy may lie in their multifunctionality. α-GalCer-activated iNKT cells can rapidly and simultaneously secrete large amounts of Th1 and Th2 cytokines, such as interferon-γ (IFN-γ), tumor necrosis factor- α (TNF-α), IL-4 and IL-13 [1], [8], [9] and can be induced under certain conditions to release the regulatory T cell (Treg) cytokine IL-10 and the Th17 cytokines IL-17 and IL-22 [10]–[12], [31]. This multiplicity of cytokine production, which includes cytokines with opposing or mutually-inhibitory roles in immune responses, may be counter-productive in therapeutic applications where polarized adaptive immunity is desired. For example, the antitumor activity of iNKT cells is associated with their secretion of Th1 cytokines, however, Th2/Treg cytokines released by iNKT cells may dampen antitumor immunity and even promote tumor growth [19], [22], [32]–[34]. To overcome this problem, several groups have synthesized α-GalCer analogues that can selectively skew iNKT cell responses towards Th1 [35], [36] or Th2 [37], [38].

Within iNKT cells there are distinct subsets based on CD4 and CD8 expression and most studies have focused on iNKT cells with CD4^+^CD8β^−^ (CD4^+^) and CD4^−^CD8β^−^ (CD4^−^) phenotypes, the main subsets seen in mice [1], [2]. However, it has become clear that human CD4^−^ iNKT cells in humans can be sub-classified into CD4^−^CD8α^−^β^−^ (double-negative or DN) and CD4^−^CD8α^+^ (comprising CD8α^+^β^−^ and CD4^−^CD8α^+^β^+^ cells) subsets [39]–[44]. These iNKT cell subsets are reported to have distinct immunological properties, with CD4^+^ iNKT cells releasing both Th1 and Th2 cytokines and CD8α^+^ and DN iNKT cells exhibiting Th1 phenotypes [9], [15], [39], [45]–[48] and cytotoxic activity [39]–[44]. Altered iNKT cell subset frequencies and functions have been described in humans with disease [21], [22], [24], [44], [48], [49]. Phase I clinical studies in cancer patients involving *in vivo* administration of α-GalCer or infusion of *ex vivo* expanded and activated iNKT cells have generally not considered the subset composition of iNKT cells being activated [26]–[30], which we hypothesize could play an important role in clinical outcome. In the present study we expanded iNKT cells *ex vivo* from healthy individuals and systematically compared the phenotypes, cytotoxic activities and cytokine profiles of the CD4^+^, DN and CD8α^+^ iNKT subsets. We report that all expanded iNKT cell subsets can kill CD1d^+^ target cells and release Th1 and Th2 cytokines, but CD8α^+^ iNKT cells display the most potent Th1/cytolytic activity while CD4^+^ iNKT cells release the most Th2 cytokines. All iNKT cell subsets similarly upregulate CD40L upon activation and induce cytokine secretion by DC, while only the CD4^+^ iNKT cell subset releases IL-9 and IL-10. Thus, CD8α^+^ iNKT cells may be ideal candidates for future iNKT cell-based cancer therapies, whereas the immunoregulatory properties of CD4^+^ iNKT cells could be beneficial for the treatment of inflammatory or autoimmune diseases.

## Materials and Methods

### Ethics Statement

This study was approved by the Research Ethics Committee of St. James's Hospital and the Adelaide and Meath Hospitals incorporating the National Children's Hospital (SJH/AMNCH), Dublin. Informed written consent was obtained from all study participants except when anonymised used buffy coat packs (obtained from the Irish Blood Transfusion Service) were used.

### Antibodies and flow cytometry

Fluorochrome-conjugated monoclonal antibodies (mAb) specific for human CD1d, CD3, CD4, CD8α, CD8β, CD11c, CD14, CD25, CD56, CD107a, CD154 (CD40L), CD161, HLA-DR, NKG2D, the Vα24 and Vβ11 chains that form the TCR present on iNKT cells and the complementarity-determining region 3 of the invariant Vα24Jα18 TCR chain (6B11) were obtained from BD Biosciences (Oxford, UK), Immunotools (Friesoythe, Germany), eBioscience (Hatfield, UK), Biolegend (San Diego, CA) or Beckman Coulter (Galway, Ireland). Cells were stained with mAbs in phosphate buffered saline containing 1% bovine serum albumin and 0.02% sodium azide and analysed using a CyAn ADP flow cytometer (Beckman Coulter, High Wycombe, UK) and FlowJo software (Treestar, Ashland, OR). For FoxP3 staining, the FoxP3 staining buffer kit (eBioscience) was used following manufacturer's instructions. For CD40L staining, cells were co-cultured for 6 hours with CD1d^+^ APC pulsed with α-GalCer (see below) in the presence of anti-CD40L mAb and 1 µM monensin (Sigma-Aldrich, Poole, UK) as described previously [50]. Cells were then stained with 6B11, CD4 and CD8α and analysed by flow cytometry. Single stained controls were used to set compensation parameters and fluorescence-minus-one controls were used to set gates.

### *Ex vivo* expansion of iNKT cells

Peripheral blood mononuclear cells (PBMC) were prepared from unselected buffy coat packs by density gradient centrifugation over Lymphoprep (Nycomed Pharma, Oslo, Norway). iNKT cells were enriched from PBMC by magnetic bead separation using 6B11 coated magnetic beads (Miltenyi Biotec, Bergisch-Gladbach, Germany). iNKT cells were then purified by cell sorting of CD3^+^Vα24^+^Vβ11^+^ cells using a MoFlo™ XDP Cell Sorter (Beckman Coulter). Sorted iNKT cells were expanded by culturing 1,000 iNKT cells in iNKT cell medium (RPMI 1640 containing 0.05 mM L-glutamine, 10% HyClone FCS, 1% penicillin-streptomycin, 1% fungizone 25 mM HEPES, 50 µM 2-mercaptoethanol, 1 mM sodium pyruvate, 1% non-essential amino acids mixture and 1% essential amino acids mixture; Gibco-BRL, Paisley, UK and Thermo-Scientific, Logan, UT) and stimulating them with 1 µg/ml phytohemaggluttinin-P (PHA-P; Sigma-Aldrich, Dublin, Ireland) and 250 U/ml IL-2 (R&D Systems, Abingdon, UK) in the presence of an excess (2×10^5^) irradiated allogeneic PBMC prepared from two donors. After 24 hours and again after 48 hours, medium was replaced with fresh iNKT cell medium containing 250 U/ml IL-2. Cells were expanded for a minimum of 3 weeks before being used in experiments.

### Generation of monocyte derived DC

Monocytes were enriched to >90% purity from PBMC isolated from buffy coat packs by positive selection using CD14 Microbeads (Miltenyi Biotec). The monocytes were allowed to differentiate into immature DC (iDC) by culturing them at densities of 10^6^ cells/ml using 3 ml/well of a 6-well plate (Corning, Amsterdam, Netherlands) in complete RPMI medium (RPMI 1640 containing 0.05 mM L-glutamine, 1% penicillin-streptomycin, 1% fungizone, 25 mM HEPES made with low-endotoxin fetal calf serum) containing 50 ng/ml granulocyte-macrophage colony-stimulating factor (GM-CSF) and 70 ng/ml IL-4. Cells were cultured for 6 days, replacing with fresh medium containing cytokines on day 3. On day 6, iDC were removed from the wells by aspiration using a wide-gauge Pasteur pipette and washed with warm RPMI medium. Flow cytometry was used to verify that differentiation into iDC had taken place and cells expressed HLA-DR and CD11c but not CD14.

### *In vitro* stimulation of iNKT cells with α-GalCer

iNKT cells were stimulated *in vitro* with a variety of α-GalCer-pulsed APC, including iDC, CD1d-transfected HeLa [36] or C1R [8] cells (hereafter referred to as HeLa-CD1d and C1R-CD1d) or the CD1d^+^ T cell line, Jurkat. As controls, mock-transfected HeLa and C1R cells (HeLa-mock and C1R-mock) and the CD1d^−^ cell line K562 were also used. In addition, PBMC from two consenting patients with B cell chronic lymphocytic leukemia (B-CLL) were obtained with ethical permission from the Haematology Clinic at St. James's Hospital, Dublin.

α-GalCer (KRN7000) was purchased from Funakoshi Co. Ltd, (Tokyo, Japan) and reconstituted in 100% DMSO at a concentration of 1 mg/ml followed by heating to 80°C for 2 minutes, sonication for 15 minutes and vortexing for 5 minutes. This concentrated stock was aliquoted and stored at −70°C. For use in iNKT cell assays, stock α-GalCer was thawed followed by heating to 80°C for 2 minutes, sonication for 10 minutes and vortexing for 1 minute. Dilution to the required concentration was then made in 37°C pre-warmed iNKT cell medium followed by heating to 80°C for 2 minutes, sonication for 5 minutes and vortexing for 1 minute. Required dilutions thereafter were made in 37°C pre-warmed medium followed by vigorous vortexing. The above-mentioned cell lines were pulsed for 18 hours with the appropriate concentrations of α-GalCer to allow for antigen processing and presentation. Medium was then removed before the addition of iNKT cells as described below.

### Cytotoxicity assays

Cytolytic degranulation of iNKT cells cultured with α-GalCer-pulsed APC was examined by flow cytometric analysis of CD107a expression by iNKT subsets after electronically gating on CD4^+^, DN and CD8α^+^ iNKT cells. iNKT cells and target cells were co-cultured for 4 hours at 1∶1 ratios in the presence of anti-CD107a FITC mAb and monensin (25 µM) was added after 1 hour to prevent proteolysis of the mAb conjugate upon reinternalization of CD107a.

To confirm that CD107a expression directly correlated with target cell death, the flow cytometry-based Total Cytotoxicity and Apoptosis Detection Kit (Immunochemistry Technologies, Bloomington, MN) was used to quantify target cell death. Target cells were incubated with carboxyfluorescein succinimidyl ester (CFSE) following the manufacturer' instructions and incubated with sorted iNKT cell subsets at a ratio of 5∶1 for 4 hours at 37°C with 5% CO_2_. After incubation, cells were harvested and 7-aminoactinomycin D (7-AAD), which is excluded by viable cells but can penetrate cell membranes of dying or dead cells, was added and cells were analysed immediately by flow cytometry. CFSE^+^ cells which stained positive for 7-AAD are deemed to be killed target cells.

### Analysis of cytokine secretion

To measure cytokine secretion by iNKT subsets, 10^5^ sorted CD4^+^, DN or CD8α^+^ iNKT cells were stimulated with medium alone, equal numbers of iDCs or HeLa-CD1d (pulsed with α-GalCer or vehicle) or 10 ng/ml of phorbol mristate acetate (PMA) and 1 µg/ml of ionomycin (both from Sigma-Aldrich) for 24 hours. The Human Th1/Th2/Th9/Th17/Th22 13plex FlowCytomix Multiplex kit (eBioscience) was used to measure levels of IFN-γ, TNF-α, IL-1β, IL-2, IL-4, IL-5, IL-6, IL-9, IL-10, IL-12p70, IL-13, IL-17a and IL-22 in the cell supernatants, following manufacturer's instructions. Results were acquired by flow cytometry and analysed using FlowCytomix pro 2.2 software (eBioscience). In some experiments, cell supernatants were assayed for selected cytokines by enzyme-linked imunosorbent assays (ELISA) using antibody pairs purchased from R&D Systems (Abingdon, UK).

### Intracellular analysis of cytokine production by iNKT subsets

iNKT cells were stimulated for 4 hours with equal numbers of HeLa-CD1d cells pulsed with αGalCer or vehicle as described above in the presence of brefeldin A (10 µg/ml, Sigma-Aldrich) to promote intracellular accumulation of cytokines. Cells were harvested and stained for cell surface expression of 6B11, CD4 and CD8α and intracellular expression of IFN-γ and IL-4 using fluorochrome-conjugated mAb obtained from BD Biosciences or eBioscience and analysed by flow cytometry.

### Statistical analysis

Statistical analysis was carried out using Prism GraphPad Version 5.0. The Kruskal-Wallis test was used to compare unpaired data in 3 groups and Dunn's multiple comparison tests were performed post-hoc to compare individual groups within an experiment. *, ** and *** represent p<0.05, p<0.01 and p≤0.001 respectively.

## Results

### Human CD4^+^ iNKT cells expand more readily than CD8α^+^ and DN iNKT cells in response to mitogen stimulation

**Figure 1:**
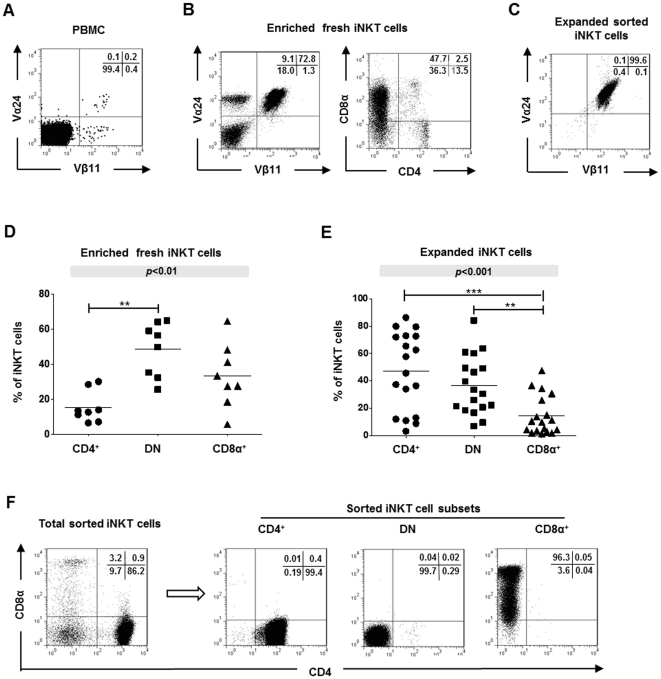
Isolation and expansion of human peripheral blood iNKT cells and analysis of CD4 and CD8α expression. **A,** Representative flow cytometry dot plot showing Vα24 and Vβ11 TCR chain expression by freshly-isolated human PBMC. **B,** Vα24 and Vβ11 TCR chain expression by PBMC after enrichment using anti-iNKT cell magnetic beads but without expansion (left panel) and CD4 and CD8α expression by gated Vα24^+^Vβ11^+^ cells (right panel). Plots are representative of PBMCs from 8 healthy donors. **C,** Vα24 and Vβ11 TCR chain expression by an expanded sorted iNKT cell line. Numbers in plots show percentages of cells in each quadrant. **D and E,** Distribution of CD4^+^, CD8α^+^ and DN cells among freshly-isolated iNKT cell lines from 8 randomly obtained healthy donors (D) and among expanded iNKT cells from 20 donors (E). Horizontal lines show means; *p* values in shaded boxes show comparisons of CD4^+^, CD8α^+^ and DN iNKT cell frequencies using the Kruskal-Wallis test; asterisks indicate significant differences between individual groups (indicated by bars) using post hoc Dunn's multiple comparison tests; **p<0.01; ***p<0.001 . **F,** Flow cytometric sorting of purified CD4^+^, CD8^+^ and DN iNKT populations from polyclonal iNKT cell lines.

The distributions of CD4^+^, DN and CD8α^+^ iNKT cells in freshly-isolated PBMC and among *in vitro* expanded iNKT cells from healthy donors were examined by flow cytometric analysis of Vα24^+^Vβ11^+^CD3^+^ cells. Since iNKT cells generally comprise <0.1% of total peripheral T cells [21], [25] (Fig. 1A), phenotypic analysis of fresh iNKT cells required prior enrichment of iNKT cells from PBMC by magnetic bead separation followed by immediate phenotyping for CD4 and CD8α (Fig. 1B). The enriched iNKT cells were then further purified by cell sorting and expanded *in vitro* with PHA-P, irradiated feeders and IL-2 for three weeks. This method resulted in a several thousand-fold increase in iNKT cell numbers with ≥98% expressing Vα24^+^Vβ11^+^ phenotypes (Fig. 1C). The mean percentages of fresh iNKT cells from 8 donors that expressed CD4^+^, DN and CD8α^+^ phenotypes were 15.6±9.0%, 48.6±15.4% and 33.5±18.1%, respectively (Fig. 1D). Virtually all iNKT cells were negative for CD8β. The 20 lines of expanded iNKT cells generated during the course of this study had highly variable distributions of the CD4^+^, DN and CD8α^+^ subsets with means of 46.2±6.1%, 37.8±4.6% and 14.5±3.1%, respectively (Fig. 1 E and F). Thus, while CD4^+^ iNKT cells generally comprise a minority of human peripheral iNKT cells, this subtype expanded more readily with mitogen stimulation in the presence of IL-2. As previously reported [44] significant numbers of double positive CD4^+^CD8α^+^ iNKT cells were also observed in freshly-isolated iNKT cell preparations (Fig. 1B). Polyclonal lines of iNKT cells were used in flow cytometry-based functional assays where electronic gating of CD4^+^, DN and CD8α^+^ expression allowed analysis of the individual subsets. For cytoxicity and cytokine secretion assays, expanded iNKT subsets were further sorted giving highly pure (>98%) populations of CD4^+^, DN and CD8α^+^ iNKT cells (Fig. 1F).

### Expanded CD4^+^, DN and CD8α^+^ iNKT cells are phenotypically distinct

**Figure 2:**
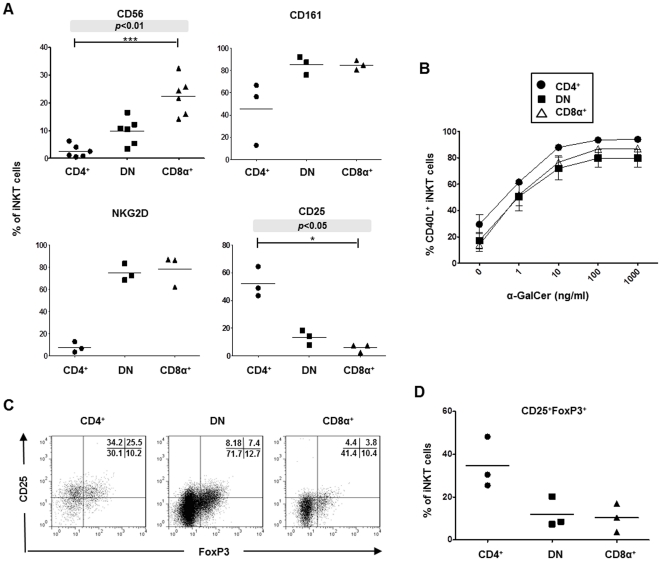
Surface phenotypes of expanded iNKT cell subsets. **A,** Percentage expression of CD56, CD161, NKG2D and CD25 by electronically-gated CD4^+^, CD8α^+^ and DN cells within expanded iNKT cell lines. Horizontal lines show means; *p* values in shaded boxes show multiple comparisons of cell frequencies using the Kruskal-Wallis test; asterisks indicate significant differences between individual groups (indicated) using post hoc Dunn's multiple comparison tests; *p<0.05; ***p<0.001. **B,** Induced cell-surface expression of CD40L by gated iNKT subsets after co-culture of iNKT cell lines with equal numbers of HeLa-CD1d pulsed with various concentrations of α-GalCer. Results show mean (±SEM) percentages of CD40L^+^ iNKT cell subsets within lines from 3 donors. **C,** Representative flow cytometry dot plots showing cell-surface CD25 and intracellular FoxP3 expression by electronically-gated CD4^+^, CD8α^+^ and DN iNKT cell subsets from 3 donors which were allowed to rest by culturing for 5 days in the absence of IL-2. Quadrants delineating positivity and negativity for CD25 and FoxP3 were set using fluorescence-minus-one control mAb staining, omitting CD25 or FoxP3. Numbers on plots show percentages of cells in each quadrant. **D,** Frequencies of CD4^+^, CD8α^+^ and DN iNKT cells within 3 iNKT cell lines that express FoxP3^+^CD25^+^ phenotypes.

iNKT lines from up to 5 healthy donors were stained with mAbs specific for the Vα24Jα18 TCR (6B11), CD4, CD8α and either the NK cell markers CD56, CD161 or NKG2D or the IL-2 receptor α-chain CD25. The proportions of gated CD4^+^, DN and CD8α^+^ iNKT cells that express these markers are shown in Figure 2A. CD56 was found to be expressed by significant numbers of CD8α^+^ iNKT cells but only by a minority of CD4^+^ iNKT cells, and on DN iNKT at intermediate frequencies. CD161 and NKG2D were present on most (>80%) CD8α^+^ and DN but on lower frequencies of CD4^+^ iNKT cells. Thus, expanded CD8α^+^ and DN iNKT cells more frequently express receptors typically found on NK cells than CD4^+^ iNKT cells. In contrast, significantly higher percentages of CD4^+^ iNKT cells expressed CD25 compared to DN and CD8α^+^ iNKT cells (Fig. 2A).

Since activated, but not resting, helper T cells express CD40L (CD154) [50], iNKT lines from 3 healthy donors were co-cultured for 6 hours with HeLa-CD1d cells pulsed with varying concentrations of α-GalCer (0–1,000 ng/ml) in the presence of monensin and anti-CD40L mAb. Figure 2B shows that CD40L expression was induced on similar percentages of CD4^+^, DN and CD8α^+^ iNKT cells in a dose-dependant manner upon activation, indicating that these iNKT cell subsets have similar potential for interacting with other cells, such as B cells and iDC.

Since CD25 was found to be expressed by significant numbers of expanded CD4^+^ iNKT cells (Fig. 2A), we next determined the frequencies of iNKT cell subsets that express the natural Treg cell phenotype, FoxP3^+^CD25^+^ (Fig. 2C). A significant proportion of CD4^+^ iNKT cells (mean 34.7±6.9%) compared to DN (12.1±4.1%) and CD8α^+^ (10.4±3.8%) expressed FoxP3^+^CD25^+^ phenotypes (Fig. 2D). However, functional studies were not carried out to test whether these cells are true regulatory iNKT cells.

### Expanded CD4^+^, DN and CD8α^+^ iNKT cells kill CD1d^+^ target cell lines and primary CD1d^+^ leukemia cells, with CD8α^+^ iNKT cells displaying the most potent cytotoxic activity

**Figure 3:**
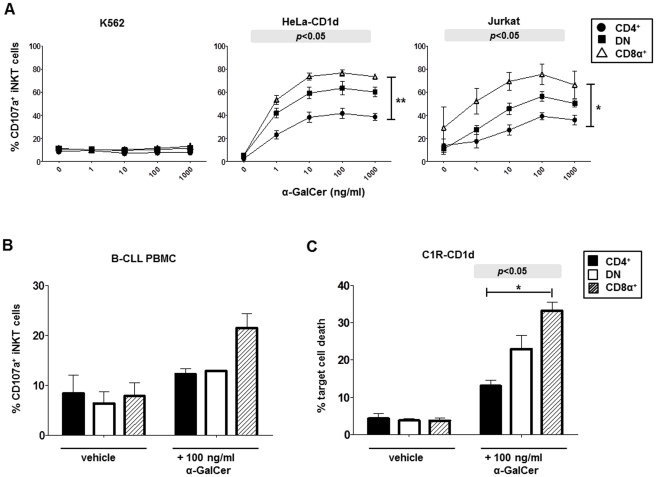
Antitumor cytotoxicity by CD4^+^, CD8α^+^, and DN iNKT cells. **A,** Expression of cell surface CD107a by iNKT cell subsets after co-culturing with CD1d^−^ K562 cells, CD1d-transfected HeLa cells or CD1d^+^ Jurkat cells, which were first pulsed for 18 hours with 0–1000 ng/ml α-GalCer. Results show mean (±SEM) percentage expression by iNKT cell subsets within 4 iNKT cell lines (3 for Jurkat). *p* values in shaded boxes show Kruskal-Wallis comparisons of CD107a expression by CD4^+^, CD8α^+^, and DN iNKT cells; asterisks indicate significant differences between CD4^+^ and CD8α^+^ iNKT cells using post hoc Dunn's multiple comparison tests; *p<0.05; **p<0.01. **B,** Surface CD107a expression by iNKT subsets in response to primary B-CLL cells pulsed with 100 ng/ml of α-GalCer. **C,** Cytolytic killing of CD1d-transfected C1R cells pulsed with 100 ng/ml α-GalCer or vehicle by highly-purified CD4^+^, DN or CD8α^+^ iNKT cells. Target cells were labelled with CFSE before addition of iNKT cells and death was then analysed by staining with 7-AAD. Data are expressed as means (±SEM) of experiments involving iNKT cell lines from 3 healthy donors. *p*<0.05 (Kruskal-Wallis); *p<0.05 comparing CD4^+^ and CD8α^+^ iNKT cells (post hoc Dunn's test).

Cytolytic degranulation by iNKT cell subsets in response to α-GalCer presented by K562, Jurkat and mock- and CD1d-transfected HeLa cells was assayed by analysis of CD107a externalization. Figure 3A shows that degranulation by CD4^+^, DN and CD8α^+^ iNKT cells only occurred when CD1d was present, since CD107a expression was undetectable when the CD1d-negative cell lines K562 cells (Fig. 3A, left panel) or HeLa-mock (not shown) were used as targets. All three iNKT cell subsets showed an α-GalCer dose-dependent increase in CD107a expression in response to the CD1d-transfected HeLa cells and the CD1d^+^ Jurkat cell line (Figure 3A, centre and right panels). Optimal CD107a expression by all 3 iNKT subsets occurred when 100 ng/ml α-GalCer was used. CD8α^+^ iNKT cells consistently expressed CD107a at higher frequencies than the other iNKT cell subsets, while CD4^+^ iNKT cells expressed this marker at the lowest frequencies (*p*<0.01 for HeLa-CD1d and Jurkat; Kruskal-Wallis).

We also investigated if iNKT cell subsets displayed cytolytic degranulation when cultured with PBMC from 2 B-CLL patients. Almost all of the lymphocytes expressed the CD19^+^CD5^+^ leukaemia phenotype and 0% and 10.1% were positive for CD1d (data not shown). iNKT cells from 3 donors exhibited undetectable degranulation when cultured with the CD1d-negative sample. When cultured with the CD1d^+^ sample, surface expression of CD107a by CD8α^+^ iNKT cells in all three iNKT cell lines was increased almost 3-fold by the addition of α-GalCer (7.8 to 21.4%) (Fig. 3B). In contrast, CD107a expression was only slightly induced on CD4^+^ and DN iNKT cells.

To confirm that CD107a expression by iNKT cell subsets correlated with target cell death, we performed cytotoxicity assays employing CFSE-labelled C1R-CD1d target cells pulsed with 100 ng/ml α-GalCer or vehicle co-cultured for 4 h with highly-purified CD4^+^, CD8α^+^ and DN iNKT cells (Figure 1C) at E∶T ratios of 5∶1. Target cell death was then assessed by staining with 7-AAD. Figure 3C shows that CD4^+^, DN and CD8α^+^ iNKT cells killed means of 13.0%, 23.0% and 33.2% of α-GalCer-primed target cells, respectively (*p*<0.05; Kruskal-Wallis). Collectively, these data indicate that CD4^+^, CD8α^+^ and DN iNKT cells are all capable of antitumor cytotoxicity and that CD8α^+^ iNKT cells consistently display the most potent cytotoxic activity, while CD4^+^ iNKT cells are the least potent and DN iNKT cells have intermediate cytolytic activity.

### Expanded CD4^+^, DN and CD8α^+^ iNKT cells have distinct cytokine secretion profiles

**Figure 4:**
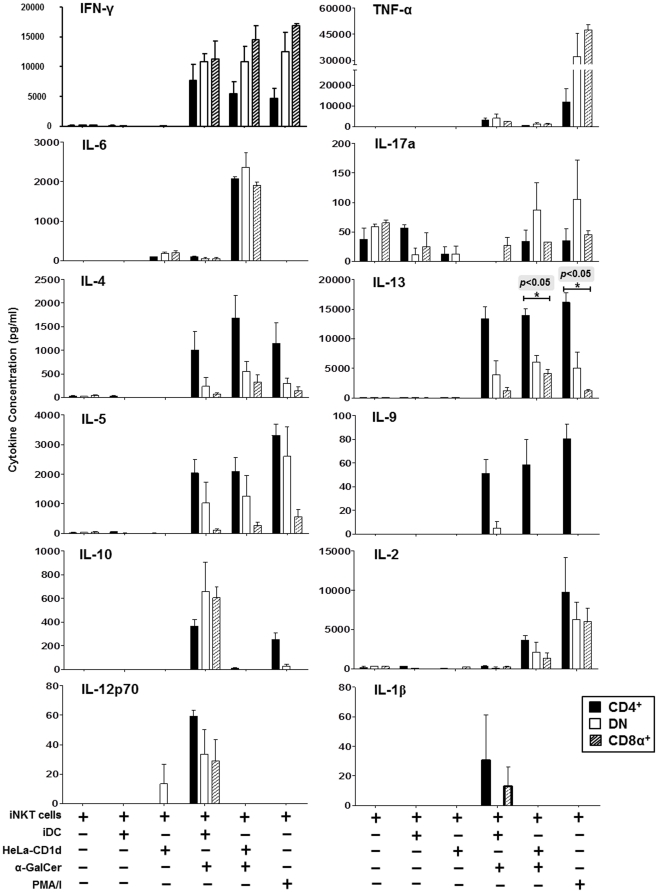
Cytokine secretion by iNKT cell subsets. Highly-purified populations of CD4^+^, DN and CD8α^+^ iNKT cells were cultured in medium alone, with iDC or HeLa-CD1d cells in the absence or presence of 100 ng/ml α-GalCer, or with PMA and ionomycin for 24 hours. Levels of IFN-γ, TNFα, IL-1β, IL-2, IL-4, IL-5, IL-6, IL-9, IL-10, IL-12p70, IL-13, IL-17a and IL-22 in cell supernatants were measured using the FlowCytomix Multiplex kit. Results show mean (±SEM) cytokine levels in experiments involving iNKT cell lines from 3 healthy donors. *p* values in shaded boxes show Kruskal-Wallis comparisons of cytokine levels released by CD4^+^, CD8α^+^, and DN iNKT cells; *p<0.05 comparing CD4^+^ and CD8α^+^ iNKT cells using post hoc Dunn's test.

Highly-purified populations of CD4^+^, DN and CD8α^+^ iNKT cells (Fig. 1F) were stimulated with medium alone, with equal numbers of iDCs or HeLa-CD1d cells (with and without 100 ng/ml α-GalCer), or with PMA and ionomycin for 24 hours. Cell-supernatants were then harvested and assayed for IFN-γ, TNFα, IL-1β, IL-2, IL-4, IL-5, IL-6, IL-9, IL-10, IL-12p70, IL-13, IL-17a and IL-22 secretion using the FlowCytomix Multiplex kit (Fig. 4). When stimulated with α-GalCer-pulsed iDC or HeLa-CD1d cells or with PMA and ionomycin, all iNKT cell subsets released IFN-γ, with CD8α^+^ iNKT cells secreting the highest levels, CD4^+^ iNKT cells secreting the lowest levels and DN iNKT cells secreting intermediate levels. α-GalCer pulsed iDCs or HeLa-CD1d cells induced low-level secretion of TNF-α, with significantly higher levels induced by PMA and ionomycin, and again, CD8α^+^ iNKT cells secreted the highest and CD4^+^ iNKT cells secreted the lowest levels. Significant IL-6 secretion was detected when all iNKT cell subsets were co-cultured with α-GalCer-pulsed HeLa-CD1d cells but not when stimulated with α-GalCer-pulsed iDC or PMA and ionomycin, suggesting that IL-6 emanated from HeLa cells, but only when cultured with iNKT cells. Slight but consistent increases in IL-17a secretion were observed when DN iNKT cells were stimulated with α-GalCer pulsed HeLa-CD1d cells or PMA and ionomycin. IL-4, IL-5 and IL-13 were predominantly secreted by activated CD4^+^ iNKT cells, while DN iNKT cells secreted intermediate levels and CD8α^+^ iNKT cells secreted the lowest levels. However, contradicting previous reports [9], [46] DN and CD8α^+^ iNKT cells were nevertheless capable of secreting these Th2 cytokines. A novel finding in the present study is the release of IL-9 by iNKT cells and only activated CD4^+^ iNKT cells were capable of secreting this cytokine under the conditions used. CD4^+^ iNKT cells were the only iNKT cell subset to secrete IL-10 after stimulation with PMA and ionomycin but not with α-GalCer pulsed HeLa-CD1d cells. However, co-cultures of iDC with all three iNKT cell subsets secreted IL-10, suggesting that this cytokine mainly emanates from iNKT-cell-matured DC. CD4^+^, DN and CD8α^+^ iNKT cells all secreted IL-2 when stimulated with α-GalCer-pulsed HeLa-CD1d cells or PMA and ionomycin, with the CD4^+^ subset secreting slightly higher levels compared to DN and CD8α^+^ iNKT cells. All three iNKT subsets stimulated IL-12p70 secretion by DC, while CD4^+^ and CD8α^+^ iNKT cells, only, induced low-level secretion of IL-1β (Fig. 4). No IL-22 was detected in supernatants of any iNKT cell subsets with any stimulation (data not shown). These data show that, under these *in vitro* conditions, α-GalCer-stimulated CD4^+^, DN and CD8α^+^ iNKT cells can release Th1 and Th2 cytokines with CD8α^+^ iNKT cells biased towards a Th1 phenotype, CD4^+^ iNKT cells predominantly secreting Th2 cytokines and DN iNKT cells exhibiting an intermediate Th1/Th2 phenotype. Low-level IL-17 is restricted to DN iNKT cells, while IL-9 and IL-10 are only released by CD4^+^ iNKT cells.

### CD4^+^, DN and CD8α^+^ iNKT cells exhibit distinct patterns of single and dual expression of IFN-γ and IL-4

**Figure 5:**
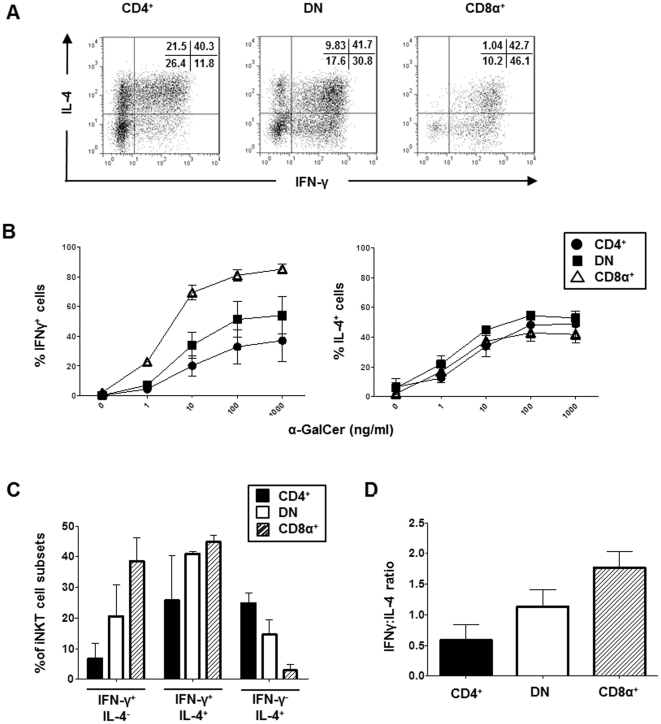
Single and dual production of IFN-γ and IL-4 production by iNKT subsets. **A,** Flow cytometry plots showing IFN-γ and IL-4 expression by iNKT cells after 4 hours of co-culture with α-GalCer pulsed HeLa-CD1d cells. 6B11^+^ cells were electronically gated followed by gating on CD4^+^, DN and CD8α^+^ cells. Plots are representative of experiments involving iNKT cell lines from 3 healthy donors. **B,** IFN-γ and IL-4 production by iNKT cell subsets stimulated with HeLa-CD1d cells pulsed with various concentrations of α-GalCer. Graphs show mean (±SEM) percentages of cells expressing IFN-γ (left) and IL-4 (right) out of iNKT cell lines from 4 healthy donors. **C,** Mean (±SEM) percentages of CD4^+^, DN and CD8α^+^ iNKT cells that expressed IFN-γ only, IFN-γ and IL-4 and IL-4 only after stimulation with HeLa-CD1d cells pulsed with 100 ng/ml α-GalCer (n = 2). **D,** Ratios of IFN-γ/IL-4-expressing CD4^+^, DN and CD8α^+^ iNKT cells after stimulation with α-GalCer-pulsed HeLa-CD1d cells (n = 2).

iNKT cells were co-cultured with α-GalCer pulsed HeLa-CD1d cells for 4 hours followed by cell-surface mAb staining of CD4 and CD8α and intracellular staining of IFN-γ and IL-4. The frequencies of IFN-γ and IL-4 single-positive and double-positive cells were determined by flow cytometry after electronically gating on the CD4^+^, DN and CD8α^+^ populations (Fig. 5A). CD8α^+^ iNKT cells displayed the highest frequencies of IFN-γ expression, with intermediate and low frequencies of DN and CD4^+^ iNKT cells, respectively, producing IFN-γ (Fig. 5B left panel). All three subsets had similar frequencies of IL-4-producing cells (Fig. 5B right panel). Interestingly, while significant numbers of all 3 iNKT cell subsets simultaneously produced IFN-γ and IL-4, single-positivity for IFN-γ was rare in the CD4^+^ iNKT cell subset and single-positivity for IL-4 was rare in CD8α^+^ iNKT cells. In contrast, stimulated DN iNKT cells had an intermediate phenotype with significant percentages producing IFN-γ or IL-4 only, in addition to double-positive IFNγ^+^IL-4^+^ cells (Fig. 5A and C). Thus, CD8α^+^ iNKT cells had the highest ratio of IFN-γ/IL-4 producing cells followed by DN iNKT cells, with CD4^+^ iNKT cells having the lowest (Fig. 5D).

## Discussion

The importance of iNKT cells in the prevention of disease and as potential therapeutic targets was first recognized with observations that mice lacking CD1d or iNKT cells are predisposed to developing cancer, autoimmune and infectious disease [6], [51] and with the discovery of α-GalCer and its beneficial effects in murine models of disease [5], [18], [19], [20]. The promising results in murine models have led to clinical trials in humans with various cancers, involving i.v. injection of α-GalCer [29] or α-GalCer-pulsed APC [26], [30]. These studies showed that iNKT-based immunotherapy is well tolerated, but the biological responses were only marginal. Subsequent therapeutic strategies involved the transfer of *ex vivo* expanded autologous iNKT cells alone or in combination with α-GalCer-pulsed APC [27], [28]. The expanded iNKT cells used in all studies were polyclonal mixtures of CD4^+^, DN and CD8α^+^ iNKT cells. Since these iNKT cell subsets differ in their immunoregulatory properties and may even be mutually-inhibitory, it may be beneficial to adoptively transfer enriched iNKT cell subsets selected for desired properties. Although previous studies have compared the effector functions of different iNKT cell subsets [9], [15], [39]–[45], [47], [48], many have been restricted to the analysis of fresh rather than expanded peripheral blood iNKT cells; many have compared CD4^+^ and CD4^−^ iNKT cells only, omitting the distinction between CD8α^−^β^−^ (DN) and CD8α^+^β^−^ (CD8α^+^) subsets; most did not use DC as APC for α-GalCer as was used in many clinical studies; and most were limited to the analysis of Th1 and Th2 cytokines only.

In the present study, we have systematically compared the phenotypes, cytolytic activities and cytokine profiles of *ex vivo* expanded human peripheral CD4^+^, DN and CD8α^+^ iNKT cells. Expanded rather than fresh iNKT cells were studied to provide information that is relevant to immunotherapy, which employs expanded iNKT cells. All 3 populations were present in PBMC, with DN iNKT cells generally being the predominant subtype followed by CD8α^+^ iNKT cells and CD4^+^ iNKT cells being the least abundant. Expansion of sorted iNKT cells from 20 randomly selected donors *in vitro* resulted in lines containing >98% Vα24^+^Vβ11^+^ cells with variable expression of CD4 and CD8α^+^. While all 3 iNKT cell subsets expanded vigorously, CD4^+^ iNKT cells were the predominant subset in our expanded lines, indicating that this subset expands most readily using our methods.

Phenotypic analysis of expanded CD4^+^, DN and CD8α^+^ iNKT cells revealed that, similar to iNKT cells in resting PBMC [9], [44], CD8α^+^ and DN iNKT cells more frequently express cell-surface receptors such as CD56, CD161 and NKG2D that are typically found on NK cells. In accordance with this NK-like phenotype and as previously reported [39]–[44], CD8α^+^ and DN iNKT cells displayed more potent cytotoxicity than CD4^+^ iNKT cells against a number of CD1d^+^ tumor cell lines, with consistently higher proportions expressing cell-surface CD107a and killing target cells. However, these cells were not capable of natural cytotoxicity, since killing was dependent on the presence of CD1d and α-GalCer. Compared to CD4^+^ and CD8α^+^ iNKT cells, DN iNKT cells displayed intermediate cytotoxic phenotypes. When PBMC from two B-CLL patients were used as targets, only CD8α^+^ iNKT cells displayed cytotoxic phenotypes. Thus, while all iNKT cell subsets possess cytolytic activity, CD8α^+^ iNKT cells are the most potent killers and are likely to be superior inducers of cell-mediated immunity. In support of this notion, CD8α^+^ iNKT cells are the most efficient at transactivating conventional CD8^+^ T cells in co-cultures with PBMC [43].

Previous studies [9], [15], [39], [45]–[48] have shown that all subsets of activated iNKT cells are capable of releasing Th1 cytokines. The present study also places CD8α^+^ iNKT cells as the most potent inducers of Th1 immunity, being capable of releasing the greatest amounts of IFN-γ and TNF-α upon stimulation with PMA and ionomycin or α-GalCer presented by iDC or HeLa-CD1d cells and being the cells that most readily produce IFN-γ in the absence of IL-4. Collectively, our data suggest that CD8α^+^ iNKT cells are superior inducers of antitumor and antiviral immunity.

In contrast to CD8α^+^ iNKT cells, we found that CD4^+^ iNKT cells release higher amounts of the Th2 cytokines IL-4, IL-5 and IL-13 upon stimulation with α-GalCer presented by iDC or HeLa-CD1d cells or with PMA and ionomycin. This predominance of Th2 cytokine secretion by CD4^+^ iNKT cells is well documented [9], [15], [39], [45], [46], [47], [48] but we also clearly show by the FlowCytomix Multiplex kit, ELISA and intracellular flow cytometry that CD8α^+^ and DN iNKT cells can also release these cytokines under the same stimulatory conditions, albeit at lower levels and lower frequencies. Analysis of dual IFN-γ and IL-4 production by activated iNKT cell subsets revealed that significant proportions of all three iNKT cell subsets could simultaneously produce both cytokines, but at markedly different ratios. Thus, iNKT cells are likely to regulate the Th1/Th2 balance of immune responses by modulating the relative levels of IFN-γ and IL-4, or IFN-γ/IL-4 ratios, rather than simply turning on or off the production of either cytokine. While potential beneficial clinical effects of each cytokine are likely to be modified by simultaneous production of the other [19], [22], [32], [33], [48], it is noteworthy that single positivity for IFN-γ was almost exclusively found in the CD8α^+^ and DN iNKT cell subsets and single positivity for IL-4 was restricted to CD4^+^ and DN iNKT cells. Thus, immunotherapy that exploits the immunoregulatory properties of iNKT cells may benefit from the use of sorted iNKT cell subsets with more polarized cytokine profiles.

We report here for the first time that human CD4^+^ iNKT cells but not CD8α^+^ nor DN iNKT cells release IL-9 in response to pharmacological or glycolipid stimulation. IL-9, a key cytokine in the recruitment and activation of mast cells, is produced by subsets of Th1, Th17 and Treg cells as well as the recently-defined Th9 subset of CD4^+^ T cells [52]. IL-9 production by a subset of non-invariant (type 2) murine CD1d-restricted CD4^+^ NKT cells is associated with IgE production [53] and antigen-induced pulmonary inflammation and mast cell infiltration [54]. In view of the reported (but disputed) infiltration of CD4^+^ iNKT cells into the lungs of humans with bronchial asthma [55], it is possible that CD4^+^ iNKT cell-derived IL-9 may play a role in the pathogenesis in some cases.

Murine and human iNKT cells have recently been reported to produce the Th17 cytokines IL-17 and IL-22 in response to α-GalCer stimulation [10], [11]. Under our stimulatory conditions human DN iNKT cells, only, were capable of releasing IL-17 but IL-22 was not detected in the supernatants of any activated iNKT cell subset. We also confirm reports [34], [39], [47], [56] that purified CD4^+^, but not DN or CD8α^+^ iNKT cells, release IL-10 and many can express CD25^+^FoxP3^+^ phenotypes, suggestive of a Treg cell phenotype. The suppressive role of IL-10 in Th1 and Th2 cell-mediated immunity will provide a further way by which iNKT cells can modulate adaptive immunity and iNKT cell-derived IL-10 is thought to suppress tumor immunity [22], [34] and protect against diabetes [57].

Rapid release of multiple cytokines is likely to have early influences on innate immune responses, however, iNKT cells can also contribute to adaptive immunity via stimulatory interactions with DC [13], [14], [58], B cells [16], [17], conventional T cells [43] and NK cells [43], [59]. Here we found that CD4^+^, DN and CD8α^+^ iNKT cells similarly upregulate CD40L upon activation and similarly induce IL-10 and IL-12 by iDC, whereas CD4^+^ and CD8α^+^ iNKT cells, only, induce IL-1β production. While all iNKT cell subsets can induce cytokine production by DC, previous studies [15], [40] have shown that CD8α^+^ iNKT cells can also kill DC, resulting in reduced IL-12 secretion and skewed Th2 immunity.

iNKT cells can be beneficial or detrimental in different disease settings [5], [6], [18]–[22], [48] resulting from cytotoxicity- and cytokine-biased immune activities. Consequently, several groups are attempting to modulate the effector profiles of iNKT cells for therapeutic application, by modifying the cytokine environment [10], [53], [60], mode of activation [11], [61] and structures of glycolipid antigens [35], [36], [37], [38], [58]. Our data show that therapeutic manipulation of iNKT cells may necessarily require the sorting of iNKT cells into functionally-distinct subsets. As well as selecting for desired effector functions, sorting of iNKT cell subsets could also allow selection of iNKT cells with distinct adhesion [46] and homing receptors [9], [42], [46], [47] that will promote optimal localization to the relevant sites.

## References

[1] Bendelac A, Savage PB, Teyton L (2007). The biology of NKT cells.. Annu Rev Immunol.

[2] Brigl M, Brenner MB (2004). CD1: antigen presentation and T cell function.. Annu Rev Immunol.

[3] Kinjo Y, Wu D, Kim G, Xing GW, Poles MA (2005). Recognition of bacterial glycosphingolipids by natural killer T cells.. Nature.

[4] Zhou D, Mattner J, Cantu C, Schrantz N, Yin N (2004). Lysosomal glycosphingolipid recognition by NKT cells.. Science.

[5] Kawano T, Cui J, Koezuka Y, Toura I, Kaneko Y (1997). CD1d-restricted and TCR-mediated activation of Vα14 NKT cells by glycosylceramides.. Science.

[6] Cui J, Shin T, Kawano T, Sato H, Kondo E (1997). Requirement for Vα14 NKT cells in IL-12-mediated rejection of tumors.. Science.

[7] Metelitsa LS, Naidenko OV, Kant A, Wu HW, Loza MJ (2001). Human NKT cells mediate antitumor cytotoxicity directly by recognizing target cell CD1d with bound ligand or indirectly by producing IL-2 to activate NK cells.. J Immunol.

[8] Exley M, Garcia J, Balk SP, Porcelli S (1997). Requirements for CD1d recognition by human invariant Vα24^+^ CD4^−^CD8^−^ T cells.. J Exp Med.

[9] Gumperz JE, Miyake S, Yamamura T, Brenner MB (2002). Functionally distinct subsets of CD1d-restricted natural killer T cells revealed by CD1d tetramer staining.. J Exp Med.

[10] Rachitskaya AV, Hansen AM, Horai R, Li Z, Villasmil R (2008). Cutting edge: NKT cells constitutively express IL-23 receptor and RORγt and rapidly produce IL–17 upon receptor ligation in an IL-6-independent fashion.. J Immunol.

[11] Goto M, Murakawa M, Kadoshima-Yamaoka K, Tanaka Y, Nagahira K (2009). Murine NKT cells produce Th17 cytokine interleukin-22.. Cell Immunol.

[12] Jiang X, Kojo S, Harada M, Ohkohchi N, Taniguchi M (2007). Mechanism of NKT cell-mediated transplant tolerance.. Am J Transplant.

[13] Kitamura H, Iwakabe K, Yahata T, Nishimura S, Ohta A (1999). The natural killer T (NKT) cell ligand α-galactosylceramide demonstrates its immunopotentiating effect by inducing interleukin (IL)-12 production by dendritic cells and IL-12 receptor expression on NKT cells.. J Exp Med.

[14] Fujii S, Shimizu K, Hemmi H, Steinman RM (2007). Innate Vα14^+^ natural killer T cells mature dendritic cells, leading to strong adaptive immunity.. Immunol Rev.

[15] Liu TY, Uemura Y, Suzuki M, Narita Y, Hirata S (2008). Distinct subsets of human invariant NKT cells differentially regulate T helper responses via dendritic cells.. Eur J Immunol.

[16] Galli G, Nuti S, Tavarini S, Galli-Stampino L, De Lalla C (2003). CD1d-restricted help to B cells by human invariant natural killer T lymphocytes.. J Exp Med.

[17] Leadbetter EA, Brigl M, Illarionov P, Cohen N, Luteran MC (2008). NK T cells provide lipid antigen-specific cognate help for B cells.. Proc Natl Acad Sci U S A.

[18] Hong S, Wilson MT, Serizawa I, Wu L, Singh N (2001). The natural killer T-cell ligand alpha-galactosylceramide prevents autoimmune diabetes in non-obese diabetic mice.. Nat Med.

[19] Crowe NY, Coquet JM, Berzins SP, Kyparissoudis K, Keating R (2005). Differential antitumor immunity mediated by NKT cell subsets in vivo.. J Exp Med.

[20] Behar SM, Porcelli SA (2007). CD1-restricted T cells in host defense to infectious diseases.. Curr Top Microbiol Immunol.

[21] Kenna T, Golden-Mason L, Porcelli SA, Koezuka Y, Hegarty JE (2003). NKT cells from normal and tumor-bearing human livers are phenotypically and functionally distinct from murine NKT cells.. J Immunol.

[22] Bricard G, Cesson V, Devevre E, Bouzourene H, Barbey C (2009). Enrichment of human CD4^+^ Vα24/Vβ11 invariant NKT cells in intrahepatic malignant tumors.. J Immunol.

[23] Molling JW, Kolgen W, van der Vliet HJ, Boomsma MF, Kruizenga H (2005). Peripheral blood IFN-γ-secreting Vα24^+^Vβ11^+^ NKT cell numbers are decreased in cancer patients independent of tumor type or tumor load.. Int J Cancer.

[24] Tahir SM, Cheng O, Shaulov A, Koezuka Y, Bubley GJ (2001). Loss of IFN-γ production by invariant NK T cells in advanced cancer.. J Immunol.

[25] Berzins SP, Smyth MJ, Baxter AG (2011). Presumed guilty: natural killer T cell defects and human disease.. Nat Rev Immunol.

[26] Chang DH, Osman K, Connolly J, Kukreja A, Krasovsky J (2005). Sustained expansion of NKT cells and antigen-specific T cells after injection of α-galactosyl-ceramide loaded mature dendritic cells in cancer patients.. J Exp Med.

[27] Motohashi S, Ishikawa A, Ishikawa E, Otsuji M, Iizasa T (2006). A phase I study of in vitro expanded natural killer T cells in patients with advanced and recurrent non-small cell lung cancer.. Clin Cancer Res.

[28] Kunii N, Horiguchi S, Motohashi S, Yamamoto H, Ueno N (2009). Combination therapy of in vitro-expanded natural killer T cells and α-galactosylceramide-pulsed antigen-presenting cells in patients with recurrent head and neck carcinoma.. Cancer Sci.

[29] Giaccone G, Punt CJ, Ando Y, Ruijter R, Nishi N (2002). A phase I study of the natural killer T-cell ligand α-galactosylceramide (KRN7000) in patients with solid tumors.. Clin Cancer Res.

[30] Ishikawa A, Motohashi S, Ishikawa E, Fuchida H, Higashino K (2005). A phase I study of α-galactosylceramide (KRN7000)-pulsed dendritic cells in patients with advanced and recurrent non-small cell lung cancer.. Clin Cancer Res.

[31] Moreira-Teixeira L, Resende M, Coffre M, Devergne O, Herbeuval JP (2011). Proinflammatory Environment Dictates the IL-17-Producing Capacity of Human Invariant NKT Cells.. J Immunol.

[32] Terabe M, Matsui S, Noben-Trauth N, Chen H, Watson C (2000). NKT cell-mediated repression of tumor immunosurveillance by IL-13 and the IL-4R-STAT6 pathway.. Nat Immunol.

[33] Moodycliffe AM, Nghiem D, Clydesdale G, Ullrich SE (2000). Immune suppression and skin cancer development: regulation by NKT cells.. Nat Immunol.

[34] Osada T, Morse MA, Lyerly HK, Clay TM (2005). Ex vivo expanded human CD4^+^ regulatory NKT cells suppress expansion of tumor antigen-specific CTLs.. Int Immunol.

[35] Schmieg J, Yang G, Franck RW, Tsuji M (2003). Superior protection against malaria and melanoma metastases by a C-glycoside analogue of the natural killer T cell ligand α-Galactosylceramide.. J Exp Med.

[36] Chang YJ, Huang JR, Tsai YC, Hung JT, Wu D (2007). Potent immune-modulating and anticancer effects of NKT cell stimulatory glycolipids.. Proc Natl Acad Sci U S A.

[37] Miyamoto K, Miyake S, Yamamura T (2001). A synthetic glycolipid prevents autoimmune encephalomyelitis by inducing TH2 bias of natural killer T cells.. Nature.

[38] Oki S, Chiba A, Yamamura T, Miyake S (2004). The clinical implication and molecular mechanism of preferential IL-4 production by modified glycolipid-stimulated NKT cells.. J Clin Invest.

[39] Takahashi T, Chiba S, Nieda M, Azuma T, Ishihara S (2002). Cutting edge: analysis of human Vα24^+^CD8^+^ NK T cells activated by α-galactosylceramide-pulsed monocyte-derived dendritic cells.. J Immunol.

[40] Ho LP, Urban BC, Jones L, Ogg GS, McMichael AJ (2004). CD4^−^CD8αα subset of CD1d-restricted NKT cells controls T cell expansion.. J Immunol.

[41] Seino K, Taniguchi M (2005). Functionally distinct NKT cell subsets and subtypes.. J Exp Med.

[42] Lin H, Nieda M, Hutton JF, Rozenkov V, Nicol AJ (2006). Comparative gene expression analysis of NKT cell subpopulations.. J Leukoc Biol.

[43] Lin H, Nieda M, Rozenkov V, Nicol AJ (2006). Analysis of the effect of different NKT cell subpopulations on the activation of CD4 and CD8 T cells, NK cells, and B cells.. Exp Hematol.

[44] Montoya CJ, Pollard D, Martinson J, Kumari K, Wasserfall C (2007). Characterization of human invariant natural killer T subsets in health and disease using a novel invariant natural killer T cell-clonotypic monoclonal antibody, 6B11.. Immunology.

[45] Lee PT, Putnam A, Benlagha K, Teyton L, Gottlieb PA (2002). Testing the NKT cell hypothesis of human IDDM pathogenesis.. J Clin Invest.

[46] Lee PT, Benlagha K, Teyton L, Bendelac A (2002). Distinct functional lineages of human Vα24 natural killer T cells.. J Exp Med.

[47] Kim CH, Butcher EC, Johnston B (2002). Distinct subsets of human Vα24-invariant NKT cells: cytokine responses and chemokine receptor expression.. Trends Immunol.

[48] Araki M, Kondo T, Gumperz JE, Brenner MB, Miyake S (2003). Th2 bias of CD4^+^ NKT cells derived from multiple sclerosis in remission.. Int Immunol.

[49] Im JS, Kang TJ, Lee SB, Kim CH, Lee SH (2008). Alteration of the relative levels of iNKT cell subsets is associated with chronic mycobacterial infections.. Clin Immunol.

[50] Chattopadhyay PK, Yu J, Roederer M (2005). A live-cell assay to detect antigen-specific CD4^+^ T cells with diverse cytokine profiles.. Nat Med.

[51] Grubor-Bauk B, Simmons A, Mayrhofer G, Speck PG (2003). Impaired clearance of herpes simplex virus type 1 from mice lacking CD1d or NKT cells expressing the semivariant Vα14-Jα281 TCR.. J Immunol.

[52] Noelle RJ, Nowak EC (2010). Cellular sources and immune functions of interleukin-9.. Nat Rev Immunol.

[53] Yoshimoto T, Min B, Sugimoto T, Hayashi N, Ishikawa Y (2003). Nonredundant roles for CD1d-restricted natural killer T cells and conventional CD4^+^ T cells in the induction of immunoglobulin E antibodies in response to interleukin 18 treatment of mice.. J Exp Med.

[54] Jones TG, Hallgren J, Humbles A, Burwell T, Finkelman FD (2009). Antigen-induced increases in pulmonary mast cell progenitor numbers depend on IL-9 and CD1d-restricted NKT cells.. J Immunol.

[55] Akbari O, Faul JL, Hoyte EG, Berry GJ, Wahlstrom J (2006). CD4^+^ invariant T-cell-receptor^+^ natural killer T cells in bronchial asthma.. N Engl J Med.

[56] Monteiro M, Almeida CF, Caridade M, Ribot JC, Duarte J (2010). Identification of regulatory Foxp3^+^ invariant NKT cells induced by TGF-β.. J Immunol.

[57] Hammond KJ, Poulton LD, Palmisano LJ, Silveira PA, Godfrey DI (1998). α/β-T cell receptor (TCR)^+^CD4^−^CD8^−^ (NKT) thymocytes prevent insulin-dependent diabetes mellitus in nonobese diabetic (NOD)/Lt mice by the influence of interleukin (IL)-4 and/or IL-10.. J Exp Med.

[58] Hogan AE, O'Reilly V, Dunne MR, Dere RT, Zeng SG (2011). Activation of human invariant natural killer T cells with a thioglycoside analogue of α-galactosylceramide.. Clin Immunol.

[59] Bricard G, Venkataswamy MM, Yu KO, Im JS, Ndonye RM (2010). α-galactosylceramide analogs with weak agonist activity for human iNKT cells define new candidate anti-inflammatory agents.. PLoS One.

[60] Lin H, Nieda M, Nicol AJ (2004). Differential proliferative response of NKT cell subpopulations to in vitro stimulation in presence of different cytokines.. Eur J Immunol.

[61] Sakuishi K, Oki S, Araki M, Porcelli SA, Miyake S (2007). Invariant NKT cells biased for IL-5 production act as crucial regulators of inflammation.. J Immunol.

